# Complete Killing of *Caenorhabditis elegans* by *Burkholderia pseudomallei* Is Dependent on Prolonged Direct Association with the Viable Pathogen

**DOI:** 10.1371/journal.pone.0016707

**Published:** 2011-03-07

**Authors:** Song-Hua Lee, Soon-Keat Ooi, Nor Muhammad Mahadi, Man-Wah Tan, Sheila Nathan

**Affiliations:** 1 School of Biosciences and Biotechnology, Faculty of Science and Technology, Universiti Kebangsaan Malaysia, UKM Bangi, Selangor, Malaysia; 2 UKM-MTDC Technology Centre, Malaysia Genome Institute, UKM Bangi, Selangor, Malaysia; 3 Department of Genetics and Department of Microbiology and Immunology, Stanford University School of Medicine, Stanford, California, United States of America; University of Washington, United States of America

## Abstract

**Background:**

*Burkholderia pseudomallei* is the causative agent of melioidosis, a disease of significant morbidity and mortality in both human and animals in endemic areas. Much remains to be known about the contributions of genotypic variations within the bacteria and the host, and environmental factors that lead to the manifestation of the clinical symptoms of melioidosis.

**Methodology/Principal Findings:**

In this study, we showed that different isolates of *B. pseudomallei* have divergent ability to kill the soil nematode *Caenorhabditis elegans*. The rate of nematode killing was also dependent on growth media: *B. pseudomallei* grown on peptone-glucose media killed *C. elegans* more rapidly than bacteria grown on the nematode growth media. Filter and bacteria cell-free culture filtrate assays demonstrated that the extent of killing observed is significantly less than that observed in the direct killing assay. Additionally, we showed that *B. pseudomallei* does not persistently accumulate within the *C. elegans* gut as brief exposure to *B. pseudomallei* is not sufficient for *C. elegans* infection.

**Conclusions/Significance:**

A combination of genetic and environmental factors affects virulence. In addition, we have also demonstrated that a *Burkholderia*-specific mechanism mediating the pathogenic effect in *C. elegans* requires proliferating *B. pseudomallei* to continuously produce toxins to mediate complete killing.

## Introduction

*Caenorhabditis elegans* is well established as a facile model to decipher the genetic factors mediating pathogen-host interactions. To date, a wide and still expanding range of pathogens have been reported to infect *C. elegans* including the Gram-negative bacteria *Pseudomonas aeruginosa* [1], *Salmonella typhimurium* [2], [3], *Serratia marcescens* [4] and *Burkholderia* species [5], [6], the Gram-positive bacteria *Staphylococcus aureus* and *Enterococcus faecalis* [7] as well as fungi such as *Drechmeria coniospora* [8] and *Yersinia pseudotuberculosis* [9]. In the case of *P. aeruginosa* and *B. cepacia*, nematode killing is growth medium-dependent and killing is associated with the proliferation of pathogenic bacteria within the digestive tract and the production of toxins. *S. typhimurium*, *S. marcescens* and *E. faecalis* are capable of stably colonizing and establishing persistent infection in the nematode intestinal tract. *D. coniospora* adheres to the surface in the region of the mouth and vulva whilst *Y. pseudotuberculosis* attaches to the cuticle in the head region and forms a biofilm which covers the mouth of *C. elegans*, thus preventing the worm from feeding.

*Burkholderia pseudomallei* is the etiologic agent for melioidosis, a disease endemic to south and east Asian countries as well as Australia [10]. Outbreaks of melioidosis in animals, including sheep, pigs, goats, cattle and dolphins have also been documented in endemic and non-endemic areas [11]. In areas where this bacterium is endemic, infection by *B. pseudomallei* has been estimated to be responsible for 20% to 30% of mortality due to septicaemia and 40% of sepsis-related mortality [12]. Human melioidosis exhibits a diverse clinical picture ranging from an asymptomatic state, to benign pneumonitis, to acute or chronic pneumonia, or to overwhelming septicaemia. The incubation period from defined inoculating events to onset of melioidosis was previously ascertained as 1–21 days [13] but the latent period has been documented to be as long as 62 years after exposure [14]. Unfortunately, treatment of *B. pseudomallei* infection is difficult as the bacterium is intrinsically resistant to many antibiotics [15].

Previous studies assessing killing kinetics of *C. elegans* by *B. pseudomallei* implicated the involvement of unidentified diffusible bacterial toxin(s) [5], [16]. They also provided support that different environmental factors could affect *C. elegans* susceptibility and/or bacterial virulence [16]. In this study, we extended the investigations by determining the virulence of *B. pseudomallei* isolated from different sources in Malaysia on *C. elegans*, and by assessing the contribution of environmental factors in nematode killing. We further ascertained, by visualization of green fluorescent protein (GFP)-expressing *B. pseudomallei*, whether killing of *C. elegans* is mediated primarily by active infection of the nematode gut or by secreted toxin(s) into the media.

## Results

### Differential susceptibility of *C. elegans* to *B. pseudomallei* isolated from different sources

**Figure 1:**
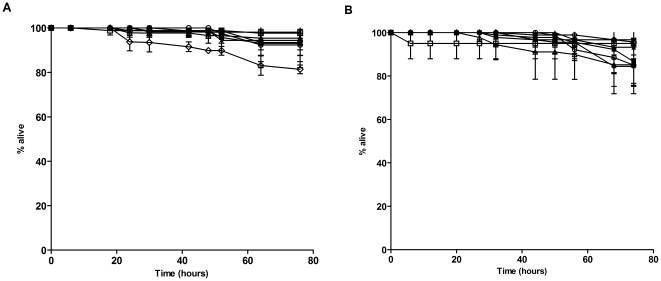
Distinct isolates of *B. pseudomallei* kill *C. elegans* with different kinetics. (A and B) One-day old Glp worms were transferred to individual *B. pseudomallei* isolates, Human PMC2000 (open diamonds), Human D286 (open triangles), Human R15 (crosses), Human H10 (open circles), Rabbit 2514 (closed squares), Sheep 4523 (closed triangles), Ostrich 9166 (closed circles) and *E. coli* OP50 (open squares). Graph shows the mean ± SD of three replicates (30 worms/replicate) from a representative of 3 independent experiments. (A) *B. pseudomallei* grown on NG medium. (B) *B. pseudomallei* grown on PG medium.

In the initial experiments conducted, we observed that the worms were laden with eggs, which in some cases hatched internally, a phenotype called “bagging of worms” [1], [2]. As the killing assay extended over the *C. elegans* generation time, progeny production may also interfere with the enumeration of surviving nematodes. Therefore, to fully eliminate any possible anomaly as a result of the bagging phenotype and laying of new progeny, we used sterile germ line proliferation-deficient (Glp) animals generated by knocking down the *cdc-25.1* gene expression [17]. The *cdc-25.1* knock-down did not affect the ability of *C. elegans* to survive infection as the TD_mean_ of Glp animals (25.61±0.93 hours) was not significantly different from wild type (Bristol N2) animals (23.62±0.92 hours) after infection by *B. pseudomallei* strain Human R15 (Logrank (Mantel-Cox) test, p = 0.06). All *B. pseudomallei* isolates tested (Table 1) were virulent on *C. elegans*: all isolates grown on NG medium caused a significant reduction in adult life span with a mean time to death (TD_mean_) of 18 hours to 56 hours compared to the worms exposed to the laboratory food source *Escherichia coli* OP50 (Figure 1A). In mortality assays on Glp animals, Sheep 4523 isolate was the most virulent, with a TD_mean_ of 18.21±0.20 hours followed by Human R15 (27.47±0.95 hours), Human PMC2000 (29.71±0.52 hours), Human D286 (31.35±0.91 hours), Ostrich 9166 (35.33±0.73 hours) and Human H10 (35.86±1.62 hours), with Rabbit 2514 being the least virulent (56.33±1.46 hours) amongst the isolates tested (Table 2). Since all *B. pseudomallei* strains were grown under identical conditions, the observed variability is likely due to intrinsic differences in genetic determinants among strains. The strains utilized in this study were previously described by Lee *et al*. [18]. All isolates (with the exception of Human PMC2000) were used to infect mice and the lethal dose 50% (LD_50_) assay was performed to determine virulence levels. To assess for possible concordance in *B. pseudomallei* virulence between the mouse and *C. elegans* models, we re-analyzed our previous data on infected mice with the Kaplan-meier analysis programme to obtain the TD_mean_ for mice infected with different isolates (Table 2). Interestingly, the bacterial virulence of a particular strain varies in different hosts, for example, the Sheep 4523 isolate, which was highly lethal to *C. elegans* had a low virulence in BALB/c mice. In contrast, the Ostrich 9166 isolate, which was less lethal to *C. elegans*, had a high virulence in BALB/c mice, suggesting that these isolates possess a distinct set of virulence factors that could potentiate different host immune responses to limit subsequent infection and further supporting that *C. elegans* may recognize a different component of pathogen-associated molecular patterns (PAMPs) than those recognized by the mammalian system. Nevertheless, virulence of the Human R15, Human D286, Human H10 and Rabbit 2514 isolates on *C. elegans* appeared concordant to their virulence in mice.

**Table 1:** Description of *B. pseudomallei* isolates utilized in this study.

Isolate	Source	Year	Reference
Sheep 4523	Sheep Farm, Ipoh, Malaysia	1998	Lee et al. 2007
Human R15	Institute for Medical Research, Kuala Lumpur, Malaysia	2005	Lee et al. 2007
Human PMC2000	Institute for Medical Research, Kuala Lumpur, Malaysia	2000	This study
Human D286	Kuala Lumpur Hospital, Malaysia	1986	Lee et al. 2007
Ostrich 9166	Veterinary Research Institute, Ipoh, Malaysia	1994	Lee et al. 2007
Human H10	Raub General Hospital, Pahang, Malaysia	1995	Lee et al. 2007
Rabbit 2514	MTDC Farm, Negeri Sembilan, Malaysia	1994	Lee et al. 2007

**Table 2:** Calculated TD_mean_ for *B. pseudomallei* isolates in infected *C. elegans* and mice.

	C. elegans		Mice	
Isolate	TD_mean_ (hours)	Ranking in order of virulence	TD_mean_ (days)	Ranking in order of virulence
Sheep 4523	18.21±0.20	1	>10	4
Human R15	27.47±0.95	2	2	1
Human PMC2000	29.71±0.52	3	NA^1^	-
Human D286	31.35±0.91	4	10	3
Ostrich 9166	35.33±0.73	5	2	1
Human H10	35.86±1.62	6	>10	4
Rabbit 2514	56.33±1.46	7	>10	4

1 Data not available.

### *C. elegans* susceptibility to *B. pseudomallei* infection is environmental factor-dependent

As the expression of bacterial virulence factors is tightly regulated by various environmental signals [19], we determined the virulence of these isolates towards *C. elegans* by growing the pathogen on plates containing peptone-glucose (PG) medium which has increased concentration of media components [20]. In contrast to the observations on NG medium (Figure 1A), worms exposed to every bacterial strain with the exception of Human H10, died significantly faster when the bacteria were grown on PG medium (Figure 1B). The time taken for nematode killing by *B. pseudomallei* grown on PG medium ranges from 5 to 35 hours. None of the worms exposed to *E. coli* OP50 died over the course of the experiment, suggesting that the difference in worm mortality is not due to intrinsic toxicity of the PG medium. Sheep 4523 (4.76±0.18 hours) and Human PMC2000 (5.87±0.23 hours) were the most virulent to the nematodes followed by Human D286 (15.21±0.79 hours), Rabbit 2514 (21.02±0.65 hours), Human R15 (21.48±0.51 hours) and Ostrich 9166 (21.73±0.70 hours), with Human H10 (34.90±1.1 hours) being the least virulent (Table 3). The data obtained above suggest that the specific dynamics of a *C. elegans- B. pseudomallei* interaction could be appreciably influenced by distinct environmental cues. As Mahajan-Miklos *et al*. [20] had previously demonstrated that expression of *P. aeruginosa* toxins involved in killing *C. elegans* is induced by high osmolarity, we repeated the experiments on Sheep 4523 and Human PMC2000 by replacing PG medium with peptone-glucose-sorbitol (PGS) medium possessing higher osmolarity. Similar killing rates were observed when *B. pseudomallei* Sheep 4523 and Human PMC2000 isolates grown on either PG or PGS medium were exposed to *C. elegans*. Sheep 4523 killed all nematodes after 12 hours post-infection on PGS medium with a TD_mean_ of 5.62±0.21 hours whereas Human PMC2000 killed all the nematodes after 20 hours post-infection on PGS medium with a TD_mean_ of 9.73±0.47 hours (Table 3).

**Table 3:** Calculated TD_mean_ for *B. pseudomallei*-infected *C. elegans* on NG, PG and PGS medium.

	TD_mean_ (hours)		
Isolate	NG	PG	PGS
Sheep 4523	18.21±0.20	4.76±0.18	5.62±0.21
Human R15	27.47±0.95	21.48±0.51	ND^1^
Human PMC2000	29.71±0.52	5.87±0.23	9.73±0.47
Human D286	31.35±0.91	15.21±0.79	ND
Ostrich 9166	35.33±0.73	21.73±0.70	ND
Human H10	35.86±1.62	34.90±1.10	ND
Rabbit 2514	56.33±1.46	21.02±0.65	ND

1 Not done.

### The role of diffusible toxin(s) in nematode killing

**Figure 2:**
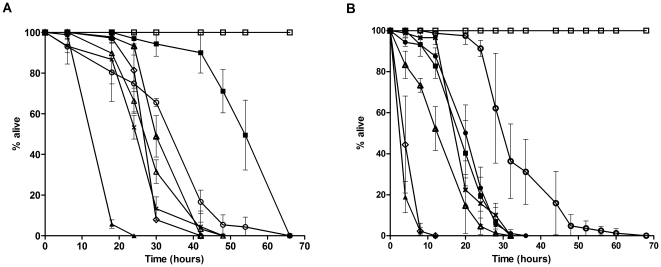
*B. pseudomallei* diffusible toxins alone are not sufficient to mediate full killing effect. (A and B) One-day old Glp worms were transferred to individual *B. pseudomallei* free culture, Human PMC2000 (open diamonds), Human D286 (open triangles), Human R15 (crosses), Human H10 (open circles), Rabbit 2514 (closed squares), Sheep 4523 (closed triangles), Ostrich 9166 (closed circles) and *E. coli* OP50 (open squares). Graph shows the mean ± SD of three replicates (30 worms/replicate) from a representative of 3 independent experiments. (A) Conditioned NG medium with grown *B. pseudomallei* on a 0.22 µm pore size sterilized nitrocellulose filter. (B) Filtered culture supernatant of *B. pseudomallei* supplemented with S-basal medium.

To determine if diffusible secreted factors contribute to *B. pseudomallei*-mediated killing of *C. elegans*, we assayed the survival of *C. elegans* exposed to culture filtrates alone. This was achieved by growing *B. pseudomallei* isolates on a 0.22 µm nitrocellulose filter placed over the media surface. As the filter pore size permits passage of secreted molecules but not the bacteria, any observed killing of the nematode population upon removal of the filter can be ascribed to the presence of diffusible toxin(s). When the filter assay was carried out on NG medium, with the exception of Human PMC2000, killing by the other isolates was negligible (Figure 2A). To validate the effect of *B. pseudomallei* secreted factors on worm mortality, we used bacterial cell-free culture filtrate and repeated the killing assay. Filtrates from all the isolates had negligible effects on worm mortality up to 76 hours of exposure (Figure 2B). Data obtained from both the filter and bacteria cell-free culture filtrate assays above demonstrated that, for all the isolates tested, secreted molecules alone were insufficient to mediate full killing. In addition, heat-killed *B. pseudomallei* also failed to cause worm mortality (data not shown). We therefore conclude that the lethal effects of *B. pseudomallei* require direct and prolonged contact between the viable bacteria and *C. elegans*.

### *B. pseudomallei* does not persistently accumulate within the *C. elegans* gut

**Figure 3:**
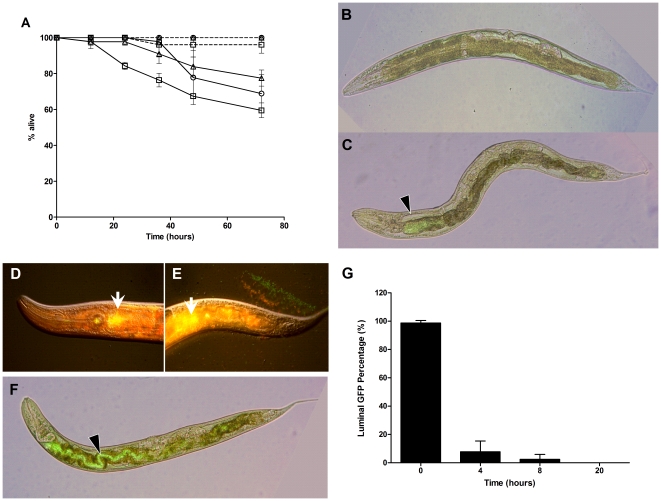
*B. pseudomallei* does not persistently accumulate within the *C. elegans* gut. (A) One-day old Glp worms fed with *B. pseudomallei* Human R15 (straight line) and *E. coli* OP50 (dashed line) for 4 hours (open triangles), 12 hours (open circles) and 20 hours (open squares) then shifted to *E. coli* OP50. Graph shows the mean ± SD of three replicates (30 worms/replicate) from a representative of 3 independent experiments. (B and C) Representative N2 worm infected with R15-GFP. These merged images show bacterial green fluorescence at the anterior intestinal lumen (black arrow head). (B) 4 hours post-infection. (C) 24 hours post-infection. (D–E) Representative *tnt-3(aj3)* mutant worm infected with R15-GFP for 6 hours and transferred to *E. coli* OP50. These merged images show bacterial fluorescence (green channel) (black arrow head) and the auto-fluorescence (red channel) (white arrow). (D) 2 hours post-shifting at the anterior intestine (E) 2 hours post-shifting at the posterior intestine. (F) Representative fully colonized *tnt-3(aj3)* mutant worm infected with R15-GFP. These merged images show bacterial green fluorescence at the anterior intestinal lumen (black arrow head). (G) Ingested R15-GFP was eradicated from the worm gut via defecation within a short period of time upon transfer to OP50 lawn. Columns represent mean ± SEM from two independent experiments.

To determine whether *B. pseudomallei* can stably colonize the *C. elegans* intestine, we performed the shifting assay with the most virulent clinical isolate (Human R15). One-day old adult worms were exposed to Human R15 for 4 hours, following which they were shifted to *E. coli* OP50 grown on NG medium. Unlike the constant exposure assay (Figure 1A), only 20% of the worms died at the 72 hour time point. The proportion of dead worms increased to 30% and 40% when worms were fed with Human R15 for 12 hours and 20 hours, respectively, prior to shifting onto *E. coli* OP50 (Figure 3A). The results indicate that brief exposure to *B. pseudomallei* was insufficient to cause a similar rate of killing to that observed when *C. elegans* was continuously in contact with Human R15 over the entire assay. Perhaps *C. elegans* is capable of overcoming a brief exposure to *B. pseudomallei*. To monitor the fate of *B. pseudomallei* within the tract of the *C. elegans* intestine upon ingestion, we repeated the experiment using GFP-expressing *B. pseudomallei* (R15-GFP). We noted that R15-GFP was unable to fully colonize the wild-type worm intestinal lumen despite continuous exposure for 4 hours up to 24 hours (Figure 3B–C). Next, we asked if R15-GFP would be able to establish an infection following initial colonization of the intestinal lumen. We increased the efficiency of initial colonization of R15-GFP bacteria in the intestinal lumen of *C. elegans* by using *tnt-3(aj3)* mutant animals, which have a defective grinder and thus allow intact bacteria to enter the intestinal lumen, but otherwise are indistinguishable from wild-type worms for immune function [21]. Using *tnt-3(aj3)*, we noted that over 50% of worms had lumens full of bacteria, as assayed by GFP fluorescence after 6 hours of exposure (data not shown). The other worms were in various degrees of partial colonization. When the partially colonized *tnt-3(aj3)* animals were shifted to plates containing only *E. coli*, we failed to detect GFP fluorescence in the gut as early as 2 hours post-shifting to OP50 (Figure 3D–E). Among the worms that were fully colonized with R15-GFP (Figure 3F), less than 10% of these animals retained luminal GFP at 4-hours post-shifting. By 20 hours after the shift to OP50, GFP fluorescence, and by extension R15-GFP, could not be detected in the intestine of any of the worms (Figure 3G). Together, these observations indicate that although *B. pseudomallei* is not able to accumulate in the *C. elegans* intestine at the gross level, nevertheless, small numbers of this bacteria are sufficient to cause a deadly effect on *C. elegans*.

## Discussion

The virulence of *B. pseudomallei* natural isolates in the *C. elegans* host model was assessed by measuring the survival of worms fed on pure cultures of these isolates. Our studies have shown differences in virulence between natural isolates of *B. pseudomallei*. These results indicated that the killing efficiency could be a result of strain specific virulence factors that directly mediate pathogenicity, as previously reported for other human pathogens such as *P. aeruginosa* [1], *S. typhimurium* [2], *B. cepacia* [5], *E. faecalis* [7] and *S. aureus* [22]. Generally, the strain-dependent pathogenicity could be explained by different genotypic and phenotypic characteristics of the *B. pseudomallei* isolates. For genetic characterization, a preliminary comparative genomics analysis of Sheep 4523, Human R15, Human PMC2000, Human D286 and Human H10 has indicated genomic differences among these five isolates predominantly within the 16 genomic islands previously reported for the *B. pseudomallei* K96243 isolate [23] (Lye and Nathan, in preparation). Variation in the pathogenic phenotype of *B. pseudomallei* natural isolates could reflect either the loss or acquisition of accessory genetic material through horizontal gene transfer events, which may confer functional diversity among individual strains. It is well established that the diverse clinical presentations observed among patients with melioidosis may be caused by genetic level variation among *B. pseudomallei* strains, including presence or absence of virulence factor genes [10]. Conversely, not all of the phenotypic traits correlated with increased mortality. The disparity in virulence levels is most likely not a simple reflection of the presence of a larger bacterial load within the worm; this is because the calculated doubling times for these isolates do not correlate with the observed TD_mean_ of the infected *C. elegans* [18]. For example, Sheep 4523 has a longer doubling time (2.3 hours) compared to Rabbit 2514 (1.9 hours) but the shorter TD_mean_ for Sheep 4523 indicates a greater level of virulence compared to Rabbit 2514. Other studies have failed to demonstrate a clear association between phenotypic behavior, especially virulence levels, with the source of the isolates (human, animal and environmental) [24], [25].

A wide range of animal models of *B. pseudomallei* infection have been developed and the host species vary widely in their pathogenesis of disease as well as their susceptibility to infection [26]. Therefore, it is not surprising that in our study some of the *B. pseudomallei* strains did not exhibit similar levels of virulence in both *C. elegans* and mice. These differences may be a consequence of the allelic constellation of the host and the microbe and their interactions with physical environments [27]. Even though we only tested a limited number of strains in this study, we do not hesitate to propose the use of *C. elegans* as a surrogate model to investigate virulent determinants of *B. pseudomallei* isolates. This is clearly reflected by the isolates that do indeed demonstrate a correlation between virulence of different *B. pseudomallei* isolates in both *C. elegans* and murine models. The impact of the complex interaction between host genetics and environmental factors also contributes to the difficulties in analyzing complex diseases in humans. In the context of *B. pseudomallei*, the symptoms of melioidosis vary widely in different host species. Therefore, it may be necessary to use different animal species to model different aspects of disease in humans [26].

As alluded to above, in addition to genetic factors, the influence of specific environmental cues on the various bacterial species is well documented. For example, *E. coli* OP50 is non-pathogenic to nematodes when grown on NG medium, but is as pathogenic as *E. faecalis* when grown on BHI agar [7]. *P. aeruginosa* grown on different media exhibited distinct modes of killing termed slow and fast killing. Slow killing resembles an infection like process over an extended period of time and correlates with the accumulation of bacteria in the nematode gut whilst fast killing is mediated by the action of diffusible toxins such as pyocyanins [1]. The high salt or hyperosmolarity conditions used in the fast killing assay are akin to those in the lung environment of cystic fibrosis patients. It is possible that the expression of particular toxins that kill *C. elegans* is induced by such conditions [19]. Our results demonstrated that *B. pseudomallei* isolates cultured on PG medium killed nematodes significantly more rapidly than on NG medium in concordance with that reported by Gan *et al*. [16]. Surprisingly, increasing the osmolarity by adding sorbitol into PG medium (PGS) did not significantly affect the dynamics of *C. elegans* killing by *B. pseudomallei*, indicating that the observed differential effects are less likely a result of change in osmolarity alone. The obtained results postulate that addition of glucose into the PG medium does contribute an additional effect towards virulence, which might explain why diabetes is a major risk factor for *B. pseudomallei* infected individuals. We have recently demonstrated that addition of different carbon sources such as glucose, sucrose and arabinose into the media did not favor an accelerated growth rate for *B. pseudomallei* (Abdul Aziz and Nathan, unpublished data). This rules out the possibility that the accelerated killing rate is associated with enhanced bacterial growth in the presence of glucose. The existence of a link between regulation of utilizable carbon sources (fructose, glucose) and expression of virulence genes has previously been proposed [28].

Previous studies have demonstrated that proliferation and accumulation of pathogens such as *P. aeruginosa, S. typhimurium* and *E. faecalis* in the intestine of *C. elegans* may constitute the primary cause of the earlier death of the worms. In marked contrast, GFP-expressing *B. pseudomallei* failed to accumulate in the worm gut to a significant extent even up to 24 hours after feeding on R15-GFP, indicating that killing of *C. elegans* by *B. pseudomallei* is not associated with the establishment and proliferation of pathogenic bacteria within the nematode gut. In addition, when we tested for persistence of *B. pseudomallei* within the *C. elegans* intestine in the transfer experiments, unlike *S. typhimurium* [2], *B. pseudomallei* fails to establish a long-lasting infection when worms are exposed to the bacteria for short periods of time. Ruling out the infection-mediated mechanism, the diffusible toxins could be the primary cause of worm killing. In contrast, the extent of killing observed via the filter assay is still significantly less than that observed in the direct killing assay (p<0.0001) implying that secreted toxin is incapable of independently causing worm mortality. Moreover, negligible nematode lethality following exposure to bacterial cell-free culture filtrates was in agreement with that reported by O'Quinn *et al*. [5] and Balaji *et al*. [29] where they demonstrated that bacteria-free culture failed to show any deleterious effect on *C. elegans*. Our findings differ from Gan *et al*. [16] who showed that in the presence of a filter, a substantial proportion (about 50%) of nematodes on NG medium were killed by *B. pseudomallei* strain KHW. This can be attributed to strain differences as previously reported [30]. Both these observations propose that neither the infection-mediated nor toxin-mediated mechanism is the primary cause of worm killing by *B. pseudomallei*. Therefore, permanent direct interaction between the host and the pathogen is crucial for a complete lethal effect i.e. the killing of worms requires living or proliferating *B. pseudomallei* to continuously produce toxins in order to mediate the full killing effect. Analysis of the *B. pseudomallei* infected worm transcriptome will reveal why the host fails to overcome infection by this pathogen and this analysis is currently on-going.

The complex interaction between a microbial pathogen and its host is the underlying basis of infectious disease. The nematode *C. elegans* has been used to provide important insights into how animals perceive and defend themselves against infection. The ability of *B. pseudomallei* to infect a wide range of animal species indicates the potential to develop a wide range of infection models. For example, in mice, the disease can range from a chronic or apparently latent infection to an acute fulminant disease depending on the route of infection, dose and mouse strain. Nevertheless, *C. elegans* is obviously limited to the study of acute infections and at the present time cannot be used to model chronic infections. This limitation underscores the necessity of a variety of complementary host-pathogen model systems in order to thoroughly understand the full complexity of *B. pseudomallei* – host interactions.

## Materials and Methods

### Bacterial isolates and nematode strains

*B. pseudomallei* isolates used are listed in Table 1. Stock cultures were stored at −80°C and routinely cultured on Ashdown media [31] at 37°C. All *B. pseudomallei* isolates were previously characterized based on biochemical tests as well as by 16S rRNA sequencing [18]. All the experiments involving *B. pseudomallei* were approved by the Universiti Kebangsaan Malaysia Animal Ethics Committee (UKMAEC) and performed in a BSL2+ level laboratory. The wild type *C. elegans* N2 and *tnt-3(aj3)* mutant strains used in this study were obtained from the Tan Laboratory at Stanford University. The nematode was propagated on nematode growth medium (NG medium) and fed on the normal food source, *E. coli* OP50 [32], at 16°C.

### Production of Glp worms

The *cdc-25.1* RNAi clone was cultured overnight in Luria-Bertani (LB) broth supplemented with 100 µg mL^−1^ ampicilin at 37°C. One hundred µL of a 25-fold –concentration liquid overnight culture was spotted onto NG medium supplemented with 1 mM Isopropyl β-D-1-thiogalactopyranoside (IPTG) plates and incubated at room temperature for 24 hours. Wild type N2 or *tnt-3(aj3)* mutant strains were made sterile by an RNAi feeding method utilizing the *cdc-25.1* RNAi clone as previously described [33]. The gene *cdc-25.1* encodes a CDC25 phosphatase homolog which affects embryonic viability and is necessary for cell proliferation in the germ line. In brief, gravid worms were laid on *cdc-25.1* RNAi plates for 4 hours and then transferred to similar plates for an additional 4 hours of egg laying. After that, gravids were removed and eggs were left to hatch and grow in the presence of *cdc-25.1* RNAi to produce sterile germ line proliferation-deficient (Glp) worms. One-day old adult hermaphrodite Glp worms reared on the *cdc-25.1* RNAi clone lawn were used in the experiments unless otherwise indicated.

### *C. elegans* survival assays

*C. elegans* survival assays were performed as previously described [1] with minor modifications. All *B. pseudomallei* isolates and *E. coli* OP50 were grown overnight in 1 mL Brain Heart Infusion (BHI) broth and LB broth respectively at 37°C. Ten µL of an overnight culture was spread over a small area on 3.5-cm NG medium or Peptone-Glucose (PG) (1% Bacto-Peptone/1% NaCl/1% glucose/1.7% Bacto-Agar) medium plates and incubated at 37°C for 24 hours. Plates were then allowed to equilibrate to room temperature for 12–24 hours before use. Thirty age-matched Glp worms were transferred to either NG medium or PG medium plates seeded with individual *B. pseudomallei* isolates and incubated at 25°C. The number of live and dead worms was scored at 4–6 hour intervals. As a negative control, *B. pseudomallei* was replaced with *E. coli* OP50. Three independent experiments were performed for each isolate.

### Filter assay

One typical colony of an individual *B. pseudomallei* isolate was inoculated into 1 mL BHI broth and incubated at 37°C overnight. Ten µL of each *B. pseudomallei* isolate overnight culture was spread onto a 0.22 µm pore size sterilized nitrocellulose filter (Whatmann) placed on 6.0-cm NG medium plates. Following incubation for 24 hours at 37°C, the filter together with the entire bacterial lawn was removed and thirty age-matched Glp worms were seeded onto the conditioned agar. Heat-killed *E. coli* OP50 was supplied to prevent starvation. Mortality of the worms was monitored at 4-hour intervals. *E. coli* OP50 was used in place of *B. pseudomallei* as the negative control. Three independent experiments were performed for each isolate.

### Bacterial cell-free culture filtrate assay

One typical colony of an individual *B. pseudomallei* isolate was inoculated into 1 mL BHI broth and incubated overnight at 37°C. The bacterial culture was centrifuged at 4000 *g* for 15 minutes at 4°C and the supernatant collected and filtered through a 0.22 µm pore size syringe membrane filter unit (Millipore). The supernatant was supplemented with 1 mL S-basal medium [34]. The assay was performed in six-well microtiter plates containing 2 mL of the supernatant and S-basal mixture. Thirty age-matched Glp worms were transferred into the conditioned liquid medium. Heat-killed *E. coli* OP50 was supplied to prevent starvation. Mortality of the worms was monitored at 4-hour intervals. *E. coli* OP50 was used in place of *B. pseudomallei* as the negative control. Three independent experiments were performed for each isolate.

### Shift assay

Approximately 100 age-matched Glp N2 worms were seeded on a *B. pseudomallei* lawn and allowed to feed. After 4, 12 and 20 hours, the worms were apportioned to three plates containing *E. coli* OP50 with 200 µg mL^−1^ tetracycline to ensure no further transfer of non-ingested *B. pseudomallei*. Worm mortality was scored every 12 hours. *E. coli* OP50 was used in place of *B. pseudomallei* as the negative control. For the shift assay involving GFP-expressing *B. pseudomallei* (Ooi and Nathan, in preparation), age matched Glp *tnt-3(aj3)* mutant worms fed on GFP-expressing *B. pseudomallei* (R15-GFP) for 6 hours were washed off from the bacterial lawns with M9 buffer and seeded on plate containing *E. coli* OP50 with 200 µg mL^−1^ tetracycline. Approximately 40 worms were placed in 100 mM levamisole on a 2% agarose pad at 0, 2, 8 and 20 hours post-shifting for microscopic examination under 400x total magnification using an upright fluorescent microscope equipped with an I3 long-pass GFP filter (Leica Microsystems, Wetzlar, Germany). Fluorescence micrographs were collected at 100x or 400x magnification using a ProgRes C10 Plus digital microscope camera (Jenoptic Laser, Jena, Germany). Each image series was captured on identical settings. Merging of microscopic photographs was done using Adobe Photoshop 7.0.

### Survival analysis

For all experiments, three replicates per trial were carried out (with a total of 90 worms) for statistical purposes. Nematodes were classified as dead when they failed to respond to touch and pharyngeal pumping was no longer observed. Worms that died as a result of being stuck to the wall were censored from the analysis. The resulting data were analyzed with *StatView* ^(R)^ 5.0.1 software (SAS Institute, Inc) and plotted using the Kaplan-Meier Cumulative Survival Plot for Time (nonparametric survival analysis). All experiments were replicated in a comparable fashion and found to produce findings similar to the data presented.
