# Correction: Complete Killing of *Caenorhabditis elegans* by *Burkholderia pseudomallei* Is Dependent on Prolonged Direct Association with the Viable Pathogen

**DOI:** 10.1371/annotation/7ae9c6d7-74ba-4dba-bc2e-4eb118559fd7

**Published:** 2011-03-22

**Authors:** Song-Hua Lee, Soon-Keat Ooi, Nor Muhammad Mahadi, Man-Wah Tan, Sheila Nathan

Figures 1 and 2 were switched in the original publication. The captions are correct.

